# Extensive bacteriocin gene shuffling in the *Streptococcus bovis*/*Streptococcus equinus* complex reveals gallocin D with activity against vancomycin resistant enterococci

**DOI:** 10.1038/s41598-020-70328-z

**Published:** 2020-08-10

**Authors:** Daragh Hill, Paula M. O’Connor, Eric Altermann, Li Day, Colin Hill, Catherine Stanton, R. Paul Ross

**Affiliations:** 1 School of Food and Nutritional Sciences, University College Cork, Cork, Ireland; 2 Teagasc Food Research Centre, Moorepark, Fermoy, Co. Cork Ireland; 3 APC Microbiome Ireland, Cork, Ireland; 4 AgResearch, Grasslands Research Centre, Palmerston North, New Zealand; 5 Riddet Institute, Massey University, Palmerston North, New Zealand; 6 School of Microbiology, University College Cork, Cork, Ireland

**Keywords:** Microbiology, Antimicrobials, Bacterial genes

## Abstract

*Streptococcus gallolyticus* LL009 produces gallocin D, a narrow spectrum two component bacteriocin with potent activity against vancomycin-resistant enterococci. Gallocin D is distinct from gallocin A, a separate two component bacteriocin produced by *S. gallolyticus*. Although the gene clusters encoding gallocin A and gallocin D have a high degree of gene synteny, the structural genes are highly variable and appear to have undergone gene shuffling with other streptococcal species. Gallocin D was analysed in laboratory-based experiments. The mature peptides are 3,343 ± 1 Da and 3,019 ± 1 Da and could be readily synthesized and display activity against a vancomycin resistant *Enterococcus* strain EC300 with a MIC value of 1.56 µM. Importantly, these bacteriocins could contribute to the ability of *S. gallolyticus* to colonize the colon where they have been associated with colorectal cancer.

## Introduction

With the rise of antibiotic resistant pathogens and the decreasing number of novel antibiotics, the search for alternative antimicrobials is of increasing importance^1^. Bacteriocins are potential antimicrobial candidates and consist of different classes of ribosomally-synthesized antimicrobial peptides which are either narrow or broad spectrum^2^. Narrow spectrum bacteriocins are of particular interest as targeted therapeutics since they could be expected to have minimal impact on resident microbiota^3,4^. Bacteriocin-producing bacteria have been isolated from a range of sources including food, skin, and the gastrointestinal tracts of both animals and humans^5^. Among the functions attributed to bacteriocins are competition, quorum sensing and host signalling^6^. They are classified into multiple types; class I are lantibiotics such as nisin which are subject to post-translational modification, and class II that are unmodified or cyclic peptides. The class II bacteriocins are divided into several subgroups^7^.

Class IIb are two-peptide bacteriocins where the two components are required for maximal activity. The structural genes encoding them are usually adjacently located, together with a gene encoding an immunity protein that protects the cell from being killed by its own bacteriocin^8^. Class IIb bacteriocin operons usually also contain an ABC transporter and an accessory protein. Both peptides are synthesised as pre-peptides with a leader sequence at the N terminal that is cleaved during export at a GG motif to produce the extracellular mature active peptide. This cleavage is performed by the ABC transporter or a peptidase which recognises the leader sequence, and transports the peptide across the cell membrane. Two-component bacteriocins require both peptides for optimal activity and both peptides interact with one another at the same target site to form one antibacterial unit. The mode of action of these bacteriocins involves the binding of the peptides to a target in the cell membrane, leading to pore formation causing leakage and cell death.

*Streptococcus gallolyticus* ssp *gallolyticus*, formerly described as *S. bovis* biotype I, is an opportunistic pathogen associated with infectious endocarditis (IE) and colorectal cancer (CRC)^9^. It is hypothesized that altered conditions in the colon associated with cancer provide an environment in which *S. gallolyticus* is able to thrive and colonise. This ability is linked to the production of a two component bacteriocin gallocin, for clarification referred to as gallocin A. In one study, using a murine model of CRC, colonisation of tumour bearing mice by *S. gallolyticus* UCN34 was mediated by production of gallocin A encoded by two structural genes, *gallo_2020* and *gallo_2021*, hereafter designated *gllA1* and *gllA2*^10^. The bacteriocin is active against enterococci, potentially creating a niche in the colon for *S. gallolyticus* colonisation. An *S. gallolyticus* UCN34 in which *gllA1* and *gllA2* were knocked out lacked this colonisation advantage in tumour bearing mice. In this study, we describe a strain of *S. gallolyticus* which harbours distinct structural genes in place of *gllA1* and *gllA2*. These structural genes are similar to genes encoding a bacteriocin produced by *Streptococcus infantarius* ssp *infantarius* (*Sii*)^11^*.*

*Streptococcus gallolyticus* and *S. infantarius* are both members of the *Streptococcus bovis*/*Streptococcus equinus* complex (SBSEC) which is divided into seven subspecies; *S. gallolyticus*, *S. infantarius*, *S. gallolyticus* ssp *macedonicus* (*Sgm*),* S. gallolyticus* ssp *pasteurianus* (*Sgp*), *Streptococcus lutetiensis*, *Streptococcus alactolyticus*, and *S. equinus*^12,13^. SBSEC members have been isolated from human, animal and food sources, with an estimated carriage rate of 5% in healthy adults. *S. gallolyticus* has been linked with IE and CRC in humans, while *S. infantarius* is found in fermented foods, predominantly in sub-Saharan Africa^14–18^. *S. infantarius* has also been linked to CRC in an African cohort, although this did not correlate to consumption of traditional fermented food containing species of *S. infantarius*. This suggests a separation between commensal, opportunistic pathogen, and food lineages^19,20^. The SBSEC genomes have been studied for their ability to colonise in their associated niche, for example some strains such as *S. gallolyticus* retain tannin degrading abilities associated with survival in ruminant animals^18,21,22^. Other strains such as *S. infantarius* and *S. gallolyticus* ssp *pasteurianus* have lost this ability and have undergone a genome reduction associated with an adaption to the dairy environment^18^. This has been discussed at length for members of the SBSEC, and it has been suggested that isolation source is associated with strain lineage^23,24^. *S. gallolyticus* strains have retained the largest genomes and most diverse functional capabilities and have been found in both healthy individuals and patients with underlying disease. It has been found in 74% of CRC patients and preferentially associates with tumour tissue^9,25^.

This study analysed class IIb bacteriocins in *S. gallolyticus* and *S. infantarius* and highlights the ability of these strains to adapt to their niche. Based on the discovery of bacteriocin structural genes in our new isolate *S. gallolyticus* LL009, we completed an in silico screen on available sequences of other SBSEC members. This analysis revealed that a similar bacteriocin operon is present in each *S. gallolyticus* strain analysed, but *S. gallolyticus* LL009 contains distinct structural and immunity genes. The bacteriocin, termed gallocin D, is a variant of a bacteriocin produced by another dairy isolate *S. infantarius* LP90^11^. A similar bacteriocin operon is also present in members of the related *S. infantarius*. The bacteriocin produced by *S. gallolyticus* LL009, that we designate gallocin D, was synthesized and analysed for activity against related *S. gallolyticus* strain and other clinically-relevant pathogens. It was found to have activity against clinically important pathogens, including *Streptococcus pneumoniae*, vancomycin resistant enterococci and another *S. gallolyticus* strain.

## Results

### Identification of the bacteriocin produced by *Streptococcus gallolyticus* LL009

**Figure 1:**
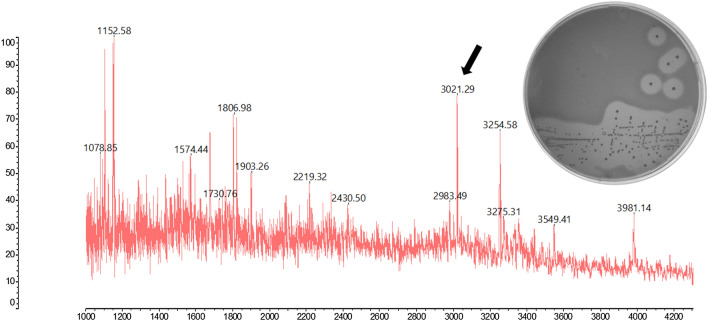
Detection of the 3,021.29 Da gallocin D2 peptide by MALDI-TOF MS from colonies on a plate. Inset shows the antimicrobial activity of *S. gallolyticus* LL009 against *L. bulgaricus* LMG6901.

*Streptococcus gallolyticus* LL009 (*Sgg* LL009) was isolated from raw goat milk sourced in New Zealand; milk samples were stored at − 20 °C until processing. The strain was initially isolated by streaking 10 µl of milk onto *Streptococcus thermophilus* selective agar that was incubated at 42 °C for 24 h. To test for bacteriocin production, individual isolates were streaked onto BHI agar and incubated overnight at 37 °C, aerobically. When overlaid with *Lb. delbrueckii* ssp *bulgaricus* LMG 6,901, zones of inhibition were observed around the colonies of *Sgg* LL009 (Fig. 1). No antimicrobial activity was observed when a cell-free supernatant was tested by well diffusion assay, but a zone of clearing was observed when cell-containing broth was used in this assay. Inhibitory activity was restored to the cell free supernatant when Tween80 was added. We designated this bacteriocin activity as gallocin D, pending further analysis.

*Sgg* LL009 was tested for the production of capsular exopolysaccharide (EPS), a feature of some *S. gallolyticus* strains. Colonies were positive for the loop touch test and were white on ruthenium red supplemented medium, indicating a ropy type EPS^26,27^. On sucrose supplemented medium, a large amount of mucous type EPS was produced.

*Sgg* LL009 was not completely resistant to any antibiotic tested (Table 1). It is non-proteolytic in that no zones of clearing were observed surrounding colonies on 10% (w/v) reconstituted skim milk (RSM). A green colouration was observed surrounding cells on blood agar for hemolysis testing, indicative of alpha hemolysis. Alpha hemolysis is defined as bruising of the red blood cells and not true lysis.

**Table 1:** Antibiotic resistance results.

Antibiotic	*S. gallolyticus* LL009 profile			EFSA *S. thermophilus*	EFSA other Gram +ve
Gentamicin	8	8	8	32	4
Kanamycin	128	128	128	64	16
Streptomycin	64	64	64	64	8
Neomycin	32	32	32	n/a	n/a
Tetracycline	0.5	0.5	0.5	4	2
Erythromycin	0.06	0.03	0.03	2	0.5
Clindamycin	0.12	0.06	0.06	2	0.25
Chloramphenicol	2	2	2	4	2
Ampicillin	0.25	0.25	0.25	2	1
Penicillin	0.12	0.12	0.12	n/a	n/a
Vancomycin	0.5	0.5	0.5	4	2
Quinupristin-dalfopristin	1	1	1	n/a	n/a
Linezolid	1	1	1	n/a	n/a
Trimethroprim	2	2	2	n/a	n/a
Ciprofloxacin	1	1	1	n/a	n/a
Rifampicin	0.25	0.12	0.25	n/a	n/a

EFSA guidelines do not have specific values for *S. gallolyticus.* Values for *S. thermophilus* and the defined values for “other Gram positive” bacteria displayed.

### In silico analysis of *Streptococcus gallolyticus* LL009

**Figure 2:**
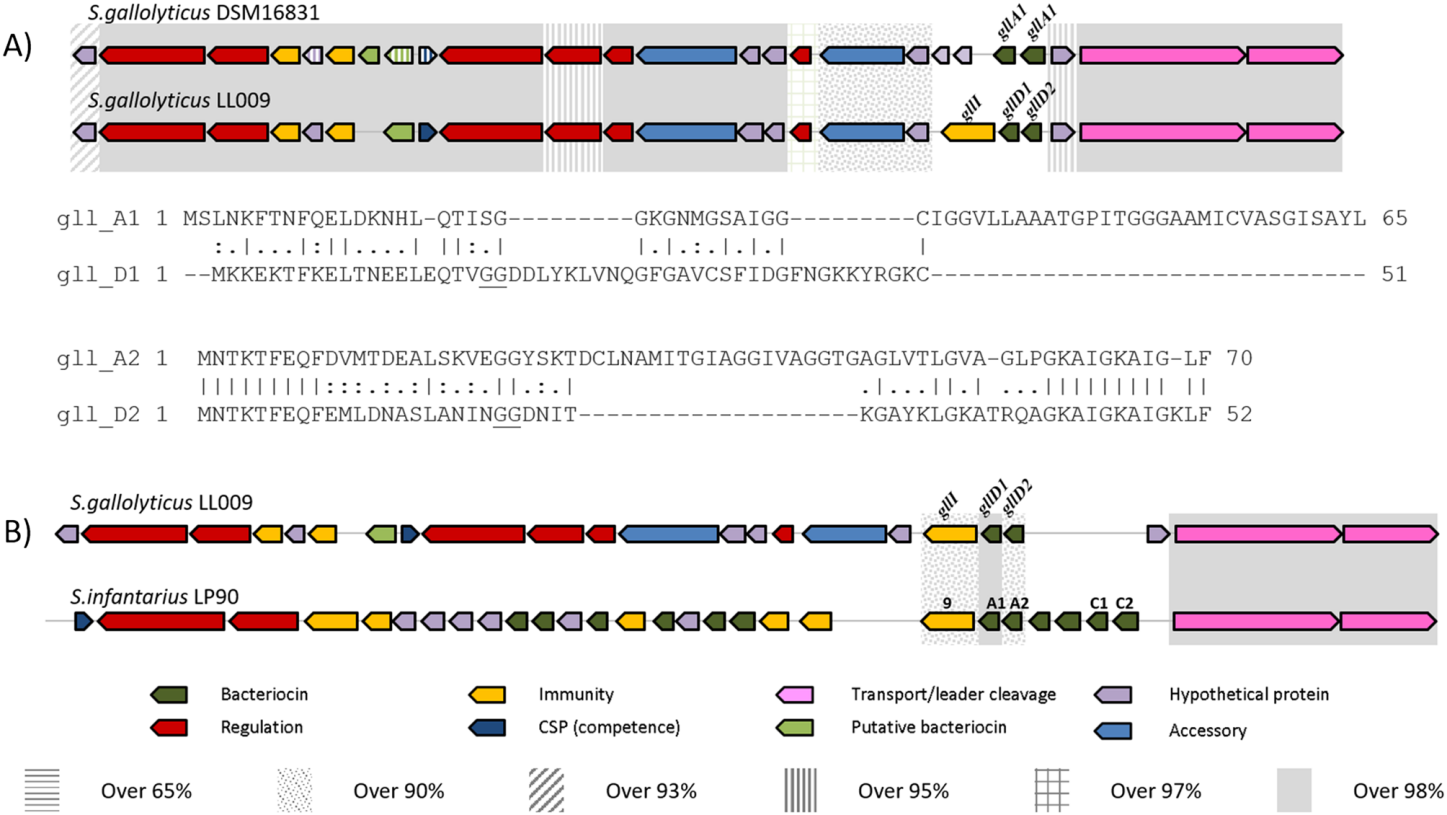
Organisation of the predicted operon encoding the bacteriocin, aligned to the operon of (**A**) type strain *S. gallolyticus* DSM 16,831, with below alignment of structural genes; *gllA2* and *gllD2* show 42% identity, *gllA1* and *gllD1* share 22% identity. (**B**) *S. infantarius* LP90 infantaricin ABCDEFG bacteriocin cluster. Genes in line with the same background colour are homologous and the amino acid percent identity is indicated.

Following draft genome sequencing of the gallocin D producer *Sgg* LL009, in silico analysis was performed using BAGEL4 and antiSMASH to identify bacteriocin-associated genes^28,29^. A single bacteriocin operon was identified in *Sgg* LL009 that consists almost entirely of genes also found in the gallocin A operon of strain *S. gallolyticus* DSM16831 (Fig. 2A). The predicted function of each gene is shown in Table 2. However, the bacteriocin structural genes (*gllD1* and *gllD2*) and that encoding the immunity protein (*gllDI*) do not have homologs in the gallocin A producing *S. gallolyticus*. These genes are homologous to genes found in the infantaricin ABCDEFG bacteriocin cluster from *S. infantarius* LP90 (Fig. 2B). The predicted structural genes in the LL009 operon are variants of infantaricin A, where *gllD1* shares 98% amino acid identity with *infA1*, while *gllD2* shares 90% amino acid identity with *infA2*. The predicted molecular weight of *gllD2*, 3,019.54 Da, is consistent with the 3,021.29 Da mass found by MALDI TOF mass spectrometry analysis (Fig. 1). This mass could not be matched to any known bacteriocin in the bactibase database.

**Table 2:** In silico analysis of the predicted function of each gene involved in bacteriocin production.

Orf	Length (aa)	Blast hit	Query cover (%)	E-value	Percent identity	Accession no
1	75	Bacteriocin, hypothetical protein [*Streptococcus gallolyticus*]	100	3.00E−45	100.00	WP_074595821.1
2	454	Two-component system, AgrA family, sensor histidine kinase AgrC [*Streptococcus gallolyticus*]	100	0	99.12	WP_074595819.1
3	255	Response regulator transcription factor [*Streptococcus gallolyticus*]	100	0	99.61	WP_081341218.1
4	97	Enterocin A Immunity [*Streptococcus gallolyticus*]	100	1.00E−63	100.00	WP_074581674.1
5	75	Hypothetical protein SGADD03_00501 [*Streptococcus gallolyticus*]	100	1.00E−45	98.67	WP_061458100.1
6	103	Enterocin A Immunity protein [*Streptococcus gallolyticus* subsp. gallolyticus ATCC 43143]	100	6.00E−68	100.00	WP_003066564.1
7	99	DUF3884 family protein putative bacteriocin [*Streptococcus gallolyticus* subsp. gallolyticus ATCC 43143]	100	2.00E−64	97.98	WP_009854998.1
8	46	Competence stimulating peptide (CSP) precursor [*Streptococcus gallolyticus*]	100	1.00E−24	100.00	AQP43123.1
9	436	Histidine kinase of the competence regulon,ComD1 [*Streptococcus gallolyticus* subsp. gallolyticus DSM 16831]	100	0	99.54	WP_058621373.1
10	243	Response regulator transcription factor [*Streptococcus gallolyticus*]	100	3.00E−178	100.00	WP_013643488.1
11	108	Multispecies: LytTR family transcriptional regulator [Streptococcus]	100	8.00E−72	99.07	WP_009855002.1
12	451	Glycosyl transferase family 2 [*Streptococcus gallolyticus* subsp. gallolyticus ATCC BAA-2069]	95	0	98.84	WP_013643489.1
13	99	Hypothetical protein BTR42_10805 [*Streptococcus gallolyticus* subsp. gallolyticus DSM 16831]	100	1.00E−66	100.00	AQP43128.1
14	60	Hypothetical protein BTR42_10810 [*Streptococcus gallolyticus* subsp. gallolyticus DSM 16831]	100	9.00E−37	100.00	WP_013643491.1
15	69	Helix-turn-helix transcriptional regulator [Streptococcus orisratti]	100	2.00E−41	98.55	WP_018375366.1
16	337	Bacteriocin biosynthesis protein [*Streptococcus gallolyticus*]	100	0	96.74	WP_077497732.1
17	71	Hypothetical protein SAMN02910295_0951 [*Streptococcus gallolyticus*]	100	3.00E−40	98.59	WP_074595803.1
18	224	InfAE-Imm [*Streptococcus infantarius* subsp. infantarius]	100	2.00E−141	92.86	WP_006531871.1
19	51	Infantaricin A1 [*Streptococcus infantarius* subsp. infantarius]	100	5.00E−28	98.04	WP_006531870.1
20	52	Infantaricin A2 [*Streptococcus infantarius* subsp. infantarius]	70	3.00E−24	90.38	WP_006531869.1
21	85	Hypothetical protein [*Streptococcus gallolyticus*]	100	3.00E−50	98.82	WP_074628192.1
22	715	ABC-type bacteriocin transporter [*Streptococcus gallolyticus*]	100	0	99.44	WP_074595798.1
23	411	Bacteriocin secretion accessory protein, partial [*Streptococcus gallolyticus*]	100	0	99.27	WP_061458107.1

**Figure 3:**
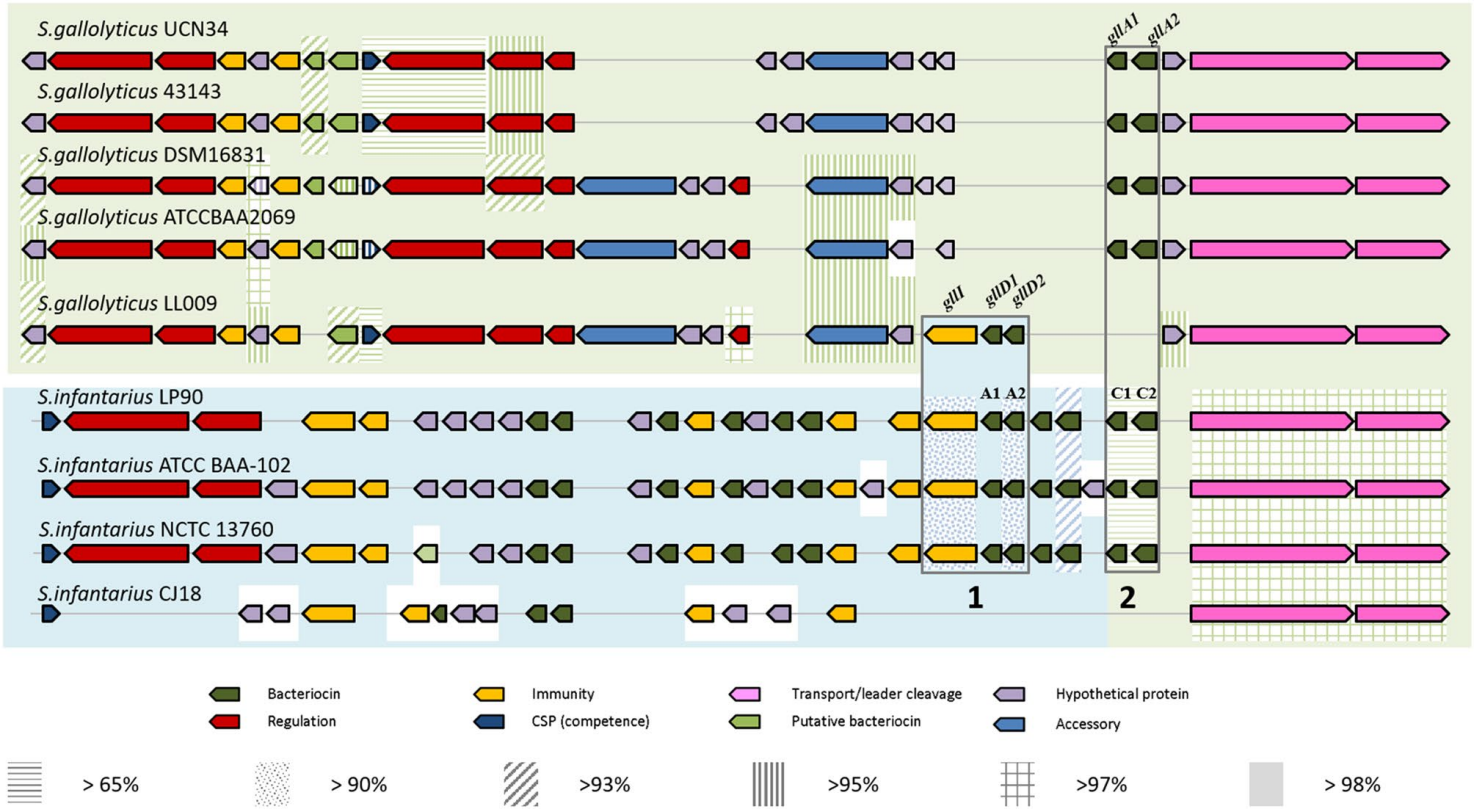
Comparison of the operons of all strains analysed. Genes in line with the same background colour are homologous (> X% amino acid sequence identity indicated), while the white background represents genes for which no homologs have been found between strains; box 1 shows the immunity protein and infantaricin A in *S. infantarius* strains and immunity protein and the gallocin D variant in *S. gallolyticus* LL009; box 2 shows potential homologs for gallocin A.

Nine strains of *S. gallolyticus* and *S. infantarius* with available genomic data (including *Sgg* LL009) were analysed for the presence of bacteriocin operons (Table 3). The ABC transporters are highly conserved in both species, with 97% amino acid identity (Fig. 3). Infantaricin A is encoded by the infantaricin ABCDEFG bacteriocin operon in *S. infantarius* LP90. Twelve structural bacteriocin genes can be identified in the operon, ten of which make up five two-peptide bacteriocins^11^. There is significant variation in the structural gene arsenal of each strain. Each previously sequenced strain of *S. gallolyticus* contained the gallocin A structural genes, *gllA1*and *gllA2*, while all *S. infantarius* strains other than CJ18 contained a homolog of infantaricin C. The infantaricin C bacteriocin genes, *infC1* and *infC2*, in *S. infantarius* LP90 have 66% and 78% identity with *gllA1* and *gllA2*, respectively. One strain of *S. gallolyticus* ssp *macedonicus* has *gllA2* next to a gene with 60% identity to *gllA1*. *Sgg* LL009 is the only *S. gallolyticus* strain that lacks a homolog for gallocin A, and instead harbours a variant of infantaricin A, here termed gallocin D.

**Table 3:** Genomes analysed in this study.

Species	Strain	NCBI reference	Source
*Streptococcus gallolyticus* ssp*. gallolyticus*	DSM 16831	NZ_CP018822.1	Koala feces
	ATCC 43143	NC_017576.1	Human blood
	ATCC BAA-2069	NC_015215.1	Human blood
	UCN34	NC_013798.1	Human blood
	LL009		Raw goat milk
*Streptococcus infantarius* ssp*. infantarius*	CJ18	NC_016826.1	Suusac fermented camel milk
	LP90	KJ475434.1	Water buffalo milk
	NCTC13760	NZ_UHFP00000000.1	Human faeces
	ATCC BAA-102	NZ_ABJK00000000.2	Human GIT

### Synthesis and spectrum of inhibition of infantaricin A and gallocin D

**Figure 4:**
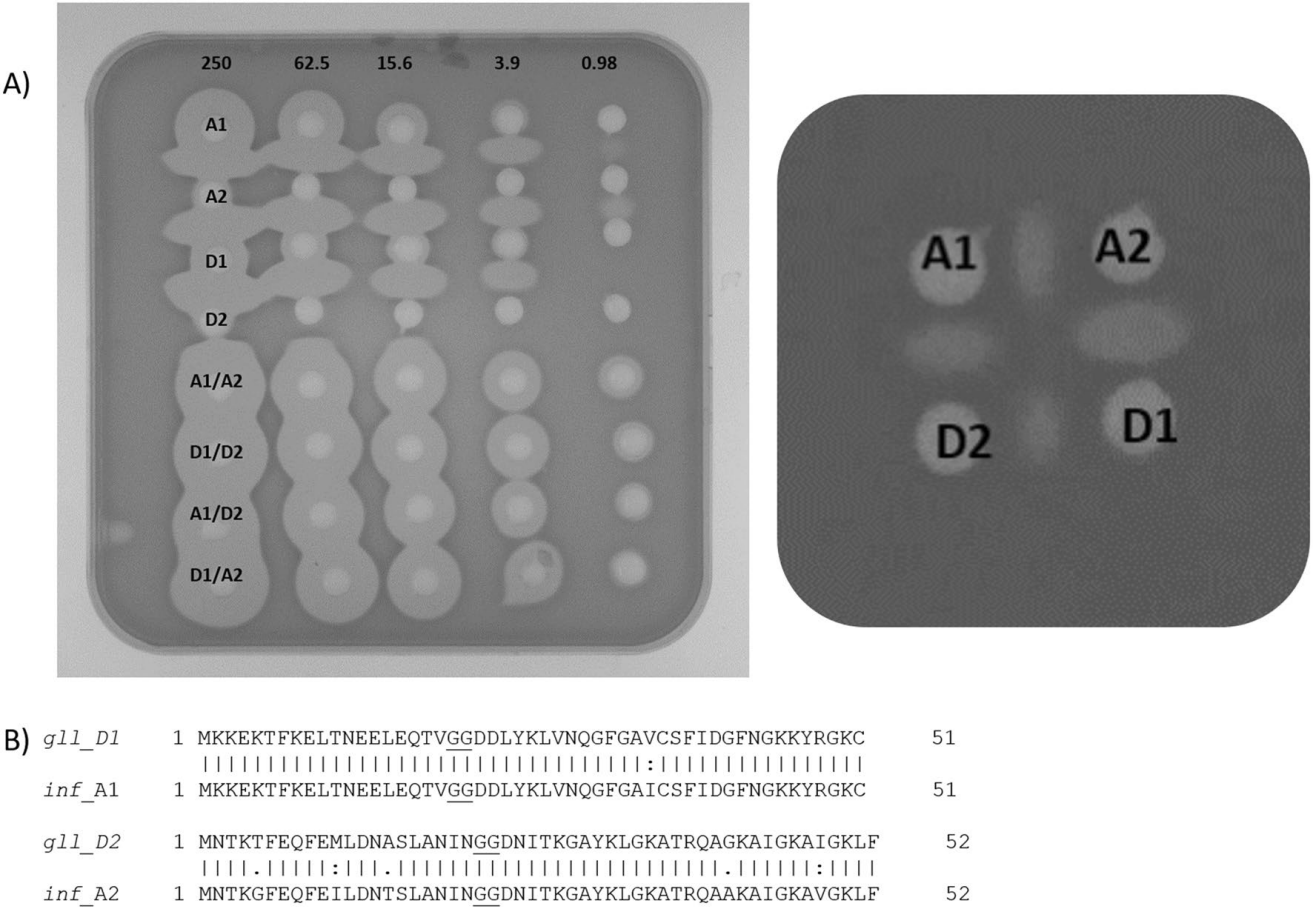
(**A**) Mixing gallocin D and infantaricin A peptides indicates that any combination of the alpha and beta peptides results in activity, concentration in µM across the top. ‘Rugby ball’ shape seen between wells indicates where the peptides meet following diffusion into the media, showing complementary activity. (**B**) Alignment of sequences of the prepeptides from both species, the double glycine cleavage point is underlined.

The gallocin D1 prepeptide encoded by *gllD1* is 51 amino acids and the D2 pre-peptide encoded by *gllD2* is 52 amino acids in length, while the active peptides are predicted to be 30 and 29 amino acids, respectively. We synthesised both D1 and D2 peptides for further characterisation, the mass of the synthesized D2 peptide matched the observed mass of 3,021 Da. The D2 peptide displays no activity in a well diffusion assay against *Lb. delbrueckii* ssp *bulgaricus*, while the D1 peptide has solo activity, which is enhanced when in a 1:1 combination with D2. When tested against pathogenic bacteria, both peptides are required for activity. The infantaricin A peptides were also synthesized and it was found that the peptides cross-complemented each other, A1/D2 or D1/A2 (Fig. 4).

A number of indicator organisms were tested to determine the spectrum of activity, using both the overlay method on a plate and a well diffusion assay using the synthesized peptide (Table 4). The bacteriocin was active against a narrow range of indicator organisms that included the clinically important pathogens *S. pneumoniae* and vancomycin resistant enterococci (VRE) strains but was inactive against other unrelated pathogenic and commensal bacteria. The minimum inhibitory concentration (MIC) of the gallocin D peptide was assessed at 1.56 µM against VRE EC300.

**Table 4:** Indicator organisms with growth conditions.

Indicator organism	Growth medium	Incubation conditions	Inhibition
*Actinomyces neuii* LMG 19524t	BHI	37°, O_2_^−^	−
*Actinomyces radingae* LMG 15960t	BHI	37°, O_2_^−^	−
*Enterococcus faecium* EC300	BHI	37°, O_2_^+^	+++
*Enterococcus faecium* EC520	BHI	37°, O_2_^+^	+++
*Enterococcus faecium* EC533	BHI	37°, O_2_^+^	+
*Enterococcus faecium* EC571	BHI	37°, O_2_^+^	++
*Escherichia coli* DPC 6,054	BHI	37°, O_2_^+^	−
*Enterococcus faecalis* E265	BHI	37°, O_2_^+^	+
*Lactobacillus delbrueckii* ssp. *bulgaricu*s LMG 6,901	MRS	37°, O_2_^−^	+++
*Lactobacillus helveticus* ATCC11454	MRS	37°, O_2_^−^	−
*Listeria innocua* DPC 1,768	BHI	37°, O_2_^+^	+
*Listeria monocytogenes* DPC 3,572	BHI	37°, O_2_^+^	−
*Salmonella enterica* ser. Typhimurium DPC 6,046	BHI	37°, O_2_^+^	−
*Staphylococcus aureus* R963	BHI	37°, O_2_^+^	−
*Streptococcus agalactiae* LMG 14,694	BHI	37°, O_2_^+^	−
*Streptococcus pnuemoniae* DSM 24,048	BHI	37°, O_2_^+^	++
*Streptococcus pnuemoniae* DSM 14,377	BHI	37°, O_2_^+^	+++
*Streptococcus pnuemoniae* DSM 20,566	BHI	37°, O_2_^+^	+++
*Streptococcus pnuemoniae* DSM 25,971	BHI	37°, O_2_^+^	++
*Streptococcus gallolyticus* DPC 6,501	BHI	37°, O_2_^+^	+++

Inhibition is represented by relative activities + 0.5–2 mm zone, ++ 2–4 mm zone, +++  > 4 mm zone + , and no inhibition −.

**Figure 5:**
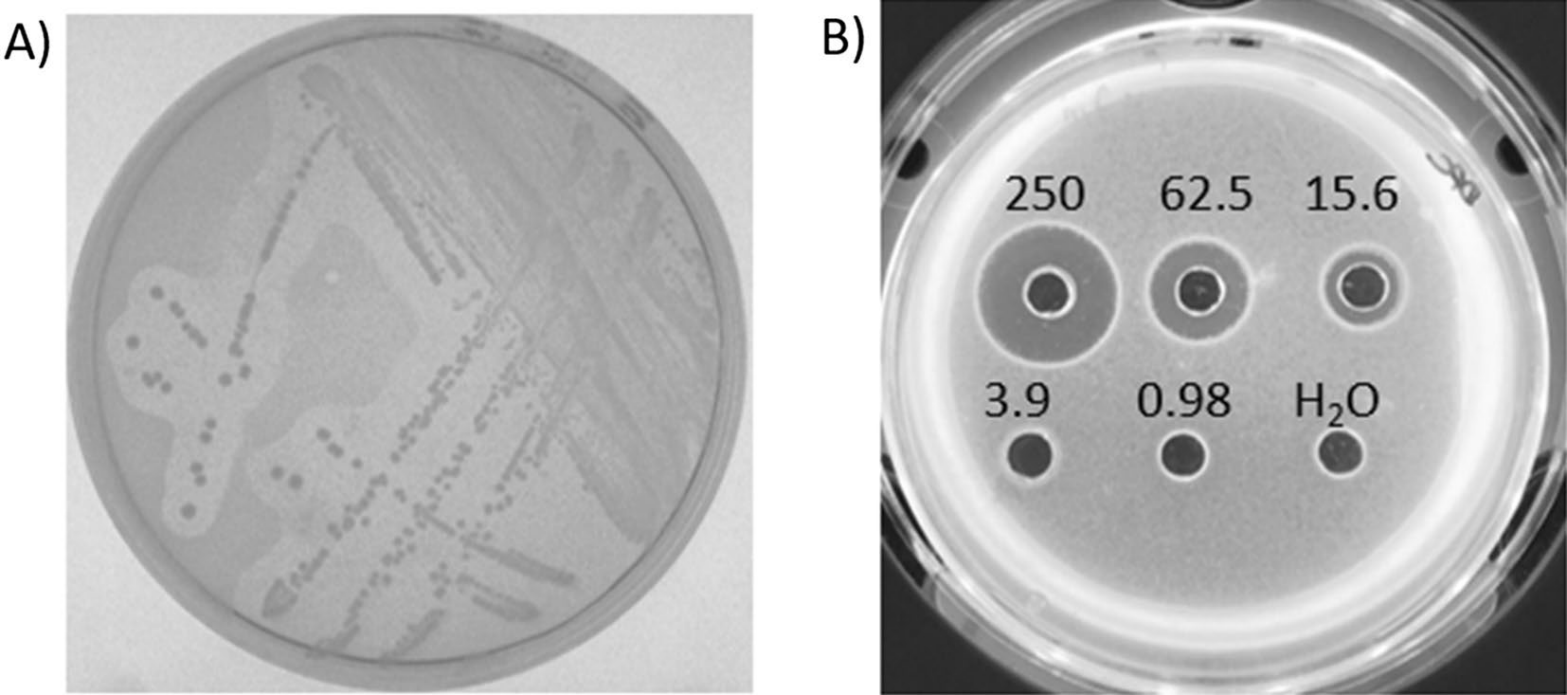
(**A**) *Sgg* LL009 colonies showing inhibition of *Sgg* DPC6501 (indicator organism). (**B**) Well diffusion assay using synthesized gallocin D against *Sgg* DPC6501, concentration in µM indicated above each well.

*Streptococcus gallolyticus* DPC6501 was isolated from a porcine jejunum in a previous study and is reported to produce a bacteriocin^30^. This strain was tested for sensitivity to *Sgg* LL009 and the synthesized gallocin D peptides. The overlay assay of *Sgg* LL009 showed zones of inhibition against *S. gallolyticus* DPC6501 and a zone of inhibition from 15 µM gallocin D peptides was observed in a well diffusion assay (Fig. 5).

### Resistance development in VRE EC300 against gallocin D

The microtitre plates from the MIC determination experiment were plated in an attempt to identify growth of VRE EC300. Following 24 h growth, VRE EC300 cells from the control and wells containing 100 µM gallocin D, the highest concentration included, were spread plated on BHI, and then sub-cultured into fresh BHI. At 24 h, the sub-cultured broths were serially diluted and plated. The bacteriocin treated cells showed no growth at any dilution, while the untreated cells reached 4.9 × 10^8^ cfu ml^−1^. At this concentration and inoculum, no colonies were found following treatment with gallocin D.

**Figure 6:**
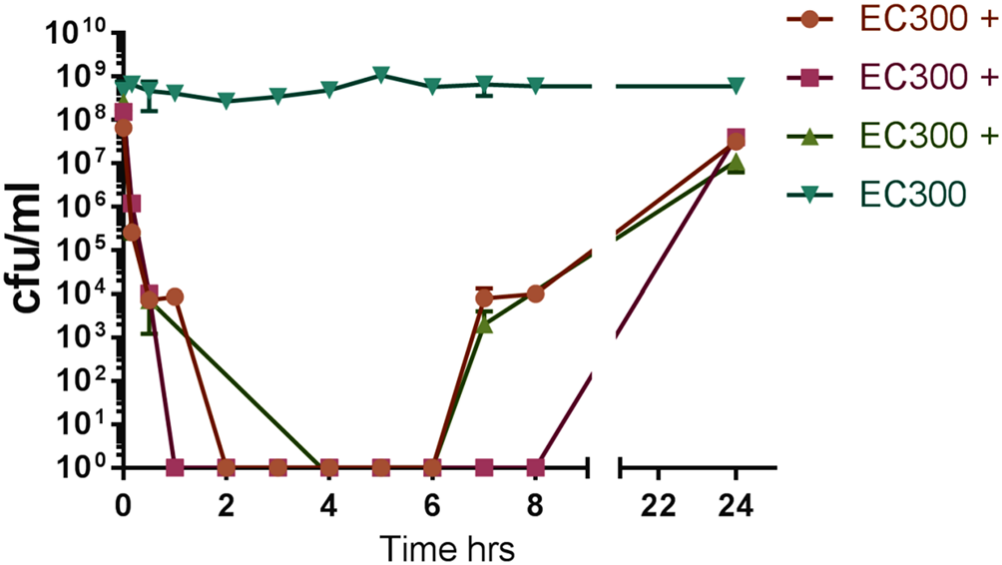
Kill curve of VRE EC300 using 15.6 µM gallocin D. EC300 + samples contain bacteriocin (each representing a biological triplicate), EC300 has no bacteriocin (control).

For the time-kill assay actively growing VRE EC300 at 10^8^ cfu ml^−1^ was treated with gallocin D at 15.6 µM, 10 × the MIC value. Within the first 4 h, VRE EC300 was undetectable in all treated broths, while the untreated control remained at 10^8^ cfu ml^−1^. At 6 h, VRE EC300 was detected in treated broths while at 24 h the treated broths were lower than the untreated control at 10^7^ cfu ml^−1^ (Fig. 6).

## Discussion

The bacteriocin operons within the *S. gallolyticus* subspecies *gallolyticus* of the SBSEC were found to be generally highly conserved, with the exception of the newly isolated *Sgg* LL009 which lacks the gallocin A structural genes and has different structural genes and a putative immunity gene at this locus. The structural genes in *Sgg* LL009 are variants of genes in an infantaricin A producing *S. infantarius* LP90 (*Sii* LP90), and its associated immunity gene. Infantaricin A is encoded by an operon with seven predicted bacteriocin structural genes, of which *infC* shows sequence homology to the gallocin A structural genes. *Sgg* LL009 is the only genome analysed found to lack genes for gallocin A/infantaricin C, and contain a variant of previously identified infantaricin A. In a BLAST search limited to the SBSEC, no gallocin D hits were found outside of the *S. gallolyticus* or *S. infantarius* species and only one potential gallocin A homolog was identified in *S. macedonicus*.

Gallocin A is a two-peptide bacteriocin which has been reported to give *S. gallolyticus* a competitive advantage in conditions found in in the gut of patients with CRC. This bacteriocin is absent from closely related species, but we identified possible homologs in *S. infantarius* strains, and *gllA2* was found in *S. gallolyticus* ssp *macedonicus* together with a gene with 60% identity to *gllA1*. High genome plasticity has been reported in the SBSEC and *S. gallolyticus* is reported to have retained the largest genome and highest functional capacity. Two human isolates of *S. infantarius*, NCTC 13,760 and ATCC BAA-102 possess the infantaricin ABCDEFG bacteriocin operon while the dairy isolate *Sii* CJ18 does not have the complete operon. *Sgg* LL009 and *Sii* LP90 were isolated from goat and water buffalo raw milk samples, respectively. In previous comparative genomics studies, high numbers of IS elements were found in these species. The organisation of the operon and high sequence identity between genes suggests that transfer of bacteriocin structural genes occurred between these strains due to adaptive pressure in the microbe-rich environment of the gut^21^. *Sgg* LL009 has unique structural genes which are produced within the gallocin D operon.

The association between *S. gallolyticus* and CRC is not fully understood^31^, but it is thought that the conditions in the colon when tumours are present provide a suitable niche for *S. gallolyticus* if competitors can be controlled with gallocin A. Gallocin A and gallocin D share similar characteristics and target organisms. Gallocin A production is enhanced in the presence of secondary bile acids, a known risk factor of CRC. The cell free supernatant of *S. gallolyticus* UCN34 shows no activity in a well diffusion assay in the absence of a detergent or secondary bile acids, a feature that is also observed for *Sgg* LL009 producing gallocin D^10^. *S. gallolyticus* mutants lacking *gllA1* and *gllA2* do not have the same colonisation advantage in tumour-bearing mice, leading to the conclusion that *S. gallolyticus* is not a causative factor of CRC but does promote its acceleration if pre-malignant tumours are present and the strain colonises. Both gallocin A and gallocin D target enterococci, with enhanced bacteriocin production in CRC conditions. This suggests that *Sgg* LL009 could well retain the colonisation advantage seen in *S. gallolyticus* UCN34 in CRC conditions, despite their distinct amino acid sequences.

The operons in the various *S. gallolyticus* strains were strikingly similar with the exception of the bacteriocin structural genes, suggesting that these bacteriocins would be subject to similar regulation and would be produced under similar conditions. Whether this strain would have a protective effect in the colon is unclear. It is likely, due to their similar target organisms and production characteristics, that this strain of *S. gallolyticus* producing gallocin D would occupy the same niche as *S. gallolyticus* producing gallocin A. Further studies are required to assess if this gallocin D producing *S. gallolyticus* is able to colonise and accelerate cancer development.

This work confirms horizontal gene transfer of bacteriocin structural genes between members of the SBSEC, which have been shown to have high genome plasticity^18^. *Sgg* LL009 and *Sii* CJ18 are both dairy isolates and both show the most “unusual” operons; the structural and immunity genes of *Sgg* LL009, and *Sii* CJ18 has the lowest number of bacteriocin structural genes of all the *Sii* strains. This hints at a role for these bacteriocins, gallocin D and infantaricin A, in streptococcal strains colonising ruminant animals. Further identification of bacteriocin producers from the SBSEC could lead to further evidence of shuffling of bacteriocin structural genes.

A further strain of bacteriocin producing *S. gallolyticus* from our culture collection, *S. gallolyticus* DPC6501, is sensitive to gallocin D, which strongly indicates that the genomic annotation of the immunity protein is correct. This is the only predicted immunity protein present in *Sgg* LL009 and absent in related *S. gallolyticus* strains and also the only immunity protein shared between *Sgg* LL009 and the infantaricin A producer. *Sgg* LL009 is not inhibited by *Sgg* DPC6501, this strain has not been sequenced, but is known to be a bacteriocin producer from previous work^30^. We hypothesise that *Sgg* LL009 is not susceptible to gallocin A, but that synthesized gallocin D could provide an alternative strategy for the control of *S. gallolyticus* infections.

The application of gallocin D is not limited to its potential role in controlling the growth of other *S. gallolyticus* strains in CRC. It is a narrow spectrum bacteriocin with potent activity against VRE, opportunistic pathogens that are particularly relevant in hospital settings, for patients who are immunocompromised and those under antibiotic treatment for endocarditis^32^. VRE can infect the urinary tract, surgical wounds or the bloodstream and are spread by direct contact. Many patients who develop VRE infections have underlying illnesses and due to antibiotic resistance this infection can lead to serious problems or fatalities^33^. The kill curve of gallocin D shows it can reduce the numbers of VRE from 10^8^ cfu ml^−1^ to undetectable levels in two hours, though the cells regrow after 8 h. Importantly, when added to growing cells at 10^5^ cfu ml^−1^, which is regarded as a clinical infection, the VRE EC300 did not recover and no resistant colonies were found. This suggests that the mode of action of this bacteriocin is related to cell contact; if the bacteriocin is present in the right ratio to cells the infection can be cleared. The MIC and resistance testing results for gallocin D show that it completely kills VRE EC300 when present at 10^5^ cfu ml^−1^, and gallocin D is present at the MIC of 1.56 µM.

## Conclusions

A combination of laboratory and in silico analyses led to the discovery and characterisation of gallocin D, a class IIb bacteriocin with activity against VRE, *S. pneumoniae*, and a related strain of *S. gallolyticus*. Gallocin D is a variant of infantaricin A produced by closely-related *S. infantarius* species. The operon is similar to those found in other *S. gallolyticus* genomes, with the exception of the structural and immunity genes. This work highlights the shuffling of bacteriocin structural genes within these closely related species. Gallocin D could be applied for treatment of VRE infections, and potentially for the control of other species of *S. gallolyticus.*

## Materials and methods

### Bacterial strains culture conditions

*S. gallolyticus* LL009 was isolated from raw goat milk produced in New Zealand on *S. thermophilus* agar at 42 °C. *S. gallolyticus* LL009 was routinely cultured under aerobic conditions at 37 °C, in brain heart infusion (BHI) medium (Oxoid Ltd., Basingstoke, Hampshire, United Kingdom), all other reagents were sourced from Sigma-Aldrich (Wicklow, Ireland) unless otherwise stated. Indicator strains used and their incubation conditions are listed in Table 3.

### Draft genome sequencing

DNA was extracted using a Genelute bacterial genomic DNA kit (Sigma) and prepared for sequencing using a Nextera XT kit (Illumina) for library preparation. DNA was quantified using a Qubit 2.0 fluorometer. Sequencing was carried out using an Illumina MiSeq platform with paired-end 2 × 300 base pair reads at the Teagasc Sequencing Centre, Teagasc Food Research Centre Moorepark. Assembly was performed de novo using SPADES and automatically annotating using GAMOLA^34^. The whole genome sequence was run through Bagel4 and antiSMASH to search for bacteriocin genes. The genome was also compared to other available *S. gallolyticus* and *S. infantarius* genomes from NCBI listed in Table 4.

### Antimicrobial activity assays

*Sgg* LL009 was grown on BHI agar and incubated overnight at 37 °C. *Sgg* LL009 was assayed against various indicator organisms, listed in Table 4 along with incubation conditions**.** Plates were overlaid with MRS agar (7.5 g L^−1^ agar) seeded with *L. bulgaricus* LMG6901 and incubated overnight at 37 °C, anaerobically. Activity was defined by a clearing in the overlay medium.

Overnight cultures were centrifuged and filtered to obtain cell free supernatant (CFS). MRS agar was seeded with an overnight culture of *L. bulgaricus* LMG6901 and wells were made. Both broth and CFS were added to the wells in 50 µl volumes. Plates were incubated overnight at 37 °C. Triton X-100 was added to CFS at 1% concentrations. All assays were performed in triplicate. The well diffusion method was repeated using synthesized gallocin D, using peptides alone and in combination (1:1).

### Minimum inhibitory concentration

Minimum inhibitory concentration (MIC) determinations of gallocin D (D1D2) were carried out in triplicate in microtitre plates as previously described^35^. Briefly, VRE EC300 was grown overnight in BHI broth at 37 °C and subcultured at 0.5% into fresh broth. The strain was grown to OD600 of 0.5 and diluted to a final concentration of 10^5^ cfu ml^−1^ in a final volume of 100 µl. Synthesized gallocin D peptides in a 1:1 ratio were made up to 100 µM concentration and serially diluted to a concentration of 0.98 µM in sterile water. The peptide solutions were added at 100 µl volumes to the VRE broth. The plate was incubated at 37 °C for 16 h, MIC was determined as the lowest concentration causing visible inhibition of growth.

### Resistance testing

In order to assess resistance development by VRE EC300, the MIC protocol was repeated. The microtitre wells containing 100 µM gallocin D and control wells were plated directly and cultured in fresh BHI broth. At 24 h, the sub-cultured broths were serially diluted and plated. The whole microtitre plate was reincubated for a further 48 h and observed for cloudiness indicative of growth.

### Kill curve

VRE EC300 was grown overnight in BHI and adjusted in fresh BHI to a final concentration of 10^8^ cfu ml^−1^. Synthesized gallocin D was added at concentration of 10 × MIC (15.6 µM) to triplicate testing broths, and sterile water added to control VRE broths. Samples were taken at multiple time points until 8 h and again at 24 h, serial diluted in MRD and plated on BHI medium. Plates were incubated for 24 h at 37 °C before colonies were enumerated.

### Colony mass spectrometry

Colony MALDI-TOF MS (Axima TOF^2^ MALDI-TOF mass spectrometer, Shimadzu Biotech) was used to determine the molecular mass of the peptides present on the surface of colonies as follows: cells were first mixed in 70% (v/v) 2-propanol/0.1% TFA (IPA) and vortex mixed, the sample was separated by centrifugation at 14,000 r.p.m and the supernatant was subsequently used for analysis. A MALDI target plate was precoated with CHCA matrix solution, 0.5 µL of the supernatant from the cell extract was then placed on the target and a final layer of matrix solution was added. Positive-ion linear mode was used to identify the peptide masses on an Axima TOF^2^ MALDI TOF mass spectrometer (Shimadzu Biotech, Manchester, UK). The masses detected were then compared to those of known bacteriocins.

### Peptide synthesis and purification

Peptides were synthesised by microwave-assisted solid phase peptide synthesis (MW-SPPS) performed on a Liberty Blue microwave peptide synthesizer (CEM Corporation. Mathews, NC, USA). Gallocin D1 was synthesized on a H-Cys(Trt)-HMPB)-ChemMatrix resin and Gallocin D2 was synthesised on H-Phe-HMPB-ChemMatrix resin (PCAS BioMAtrix Inc., Quebec, Canada). Crude peptide was purified using RP-HPLC on a Semi Preparative Jupiter Proteo C12 (10 × 250 mm, 4 µ, 90 Å) column (Phenomenex, Cheshire, UK) running acetonitrile 0.1% TFA gradients specific to the peptide of interest. Fractions containing the desired molecular mass were identified using MALDI-TOF-mass spectrometry in positive in linear mode and were pooled and lyophilized on a Genevac HT 4X lyophiliser (Genevac Ltd., Ipswich, UK).

### Growth curve

A single colony of VRE was inoculated into BHI medium and incubated at 37 °C for 16 h, and subcultured at 1% into fresh medium. Samples were taken at 0, 2, 4, 5, 6, 7, 8 and24 hours for OD_600_ and plating. OD was read in duplicate, 100 µl was serially diluted in MRD to 10^–8^ and plated on BHI agar. Plates were incubated for 24 h and enumerated. Experiments were completed in triplicate, each with technical duplicates.

### Antibiotic resistance

The MIC value of sixteen antibiotics was assessed using the VetMIC Lact-1 and Lact-2 MIC determination plates (National Veterinary Institute, Sweden). The antibiotics tested were ampicillin, penicillin, vancomycin, erythromycin, virginiamycin, tetracycline, clindamycin, chloramphenicol, kanamycin, gentamycin, streptomycin, neomycin, linezolid, rifampicin, ciprofloxacin, and trimethoprim. Briefly, colonies were resuspended in MRD at a concentration of ~ 1 × 10^8^ cfu ml^−1^ and transferred into ISO-MRS broth for a final inoculum of 5 × 10^5^ cfu ml^−1^. VetMIC plates were inoculated with 100 µl, sealed and incubated for 24 h. Evidence of growth was determined using a backlight, MIC value is the lowest concentration completely inhibiting growth. EFSA guidelines do not have specific values for *S. gallolyticus*, so values were interpreted using *S. thermophilus* values and the defined values for other Gram positive bacteria^36^.

### Exopolysaccharide screening

Multiple screening methods were used to test for ropy and non-ropy type EPS; ruthenium red agar, the loop touch test and sucrose-supplemented MRS. Ruthenium red was filter sterilised, added to cooling MRS agar at 0.08% and mixed before pouring plates27. For the loop touch test, a sterile loop was touched to a single colony and slowly pulled away. A string between the loop and colony was recorded as a positive result. 10% (w/v) sucrose and 10% (w/v) lactose supplemented MRS plates were autoclaved at 121 °C for 15 min and poured, a mucous phenotype was characterised as a positive result.

### Proteolysis

10% reconstituted skim milk (RSM) was autoclaved at 121 °C for 5 min and combined with a 3% (w/v) agar solution, autoclaved at 121 °C for 15 min, the solutions were allowed to cool to ~ 45 °C and combined 1:1, and poured into petri dishes. Previously grown *Sgg* LL009 was re-streaked onto the RSM plates and incubated at 37 °C for 48 h, plates were viewed every 24 h. Proteolysis was defined as clear zones surrounding colonies.

### Hemolysis

Tryptic Soy Agar was prepared 5% v/v sterile defibrinated horse blood was pre-warmed, added to the sterile agar and mixed well before pouring. Previously grown *Sgg* LL009 was re-streaked onto the plates and incubated for 72 h, plates were viewed every 24 h for lysis, defined as a clearing or discolouration of the agar.
